# 1-Dibenzylamino-1-de­oxy-4,5-*O*-isopropyl­idene-β-d-fructopyran­ose

**DOI:** 10.1107/S1600536810053973

**Published:** 2011-01-15

**Authors:** Shiyong Huo, Yueqing Li, Chaoyan Liang, Jihong Liu, Weijie Zhao

**Affiliations:** a School of Pharmaceutical Science and Technology, Dalian University of Technology, No. 2 Linggong Road, Ganjingzi District, Dalian 116024, People’s Republic of China

## Abstract

The title compound C_23_H_29_NO_5_, synthesized by the Amadori rearrangement of α-d-glucose with dibenzyl­amine and the ketalization, is shown to be a β-anomer. The fructopyran­ose ring adopts a chair conformation. The two benzene rings form a dihedral angle of 68.9 (1)°. In the crystal, non–classical inter­molecular C—H⋯O hydrogen bonds link the mol­ecules into a three–dimensional network.

## Related literature

For details of the synthesis of the title compound and the related ketone catalyst for asymmetric epoxidation, see: Shu *et al.* (2003 ▶); Tian *et al.* (2000 ▶, 2002 ▶).
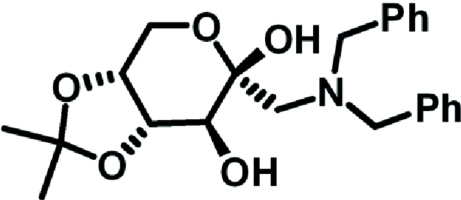


## Experimental

### 

#### Crystal data

C_23_H_29_NO_5_
*M* *_r_* = 399.47
Orthorhombic, 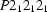

*a* = 8.328 (3) Å
*b* = 15.635 (5) Å
*c* = 16.547 (5) Å
*V* = 2154.6 (12) Å^3^
*Z* = 4
Mo *K*α radiation
μ = 0.09 mm^−1^
*T* = 293 K
0.32 × 0.26 × 0.18 mm

#### Data collection

Bruker SMART APEX CCD diffractometer
8591 measured reflections
3746 independent reflections
3078 reflections with *I* > 2σ(*I*)
*R*_int_ = 0.029

#### Refinement

*R*[*F* ^2^ > 2σ(*F* ^2^)] = 0.037
*wR*(*F* ^2^) = 0.099
*S* = 0.99
3746 reflections
264 parameters
12 restraints
H-atom parameters constrained
Δρ_max_ = 0.16 e Å^−3^
Δρ_min_ = −0.14 e Å^−3^

### 

Data collection: *SMART* (Bruker, 2005 ▶); cell refinement: *SAINT-Plus* (Bruker, 2001 ▶); data reduction: *SAINT-Plus*; program(s) used to solve structure: *SHELXS97* (Sheldrick, 2008 ▶); program(s) used to refine structure: *SHELXL97* (Sheldrick, 2008 ▶); molecular graphics: *SHELXTL* (Sheldrick, 2008 ▶); software used to prepare material for publication: *SHELXTL*.

## Supplementary Material

Crystal structure: contains datablocks I, global. DOI: 10.1107/S1600536810053973/rk2247sup1.cif

Structure factors: contains datablocks I. DOI: 10.1107/S1600536810053973/rk2247Isup2.hkl

Additional supplementary materials:  crystallographic information; 3D view; checkCIF report

## Figures and Tables

**Table 1:** Hydrogen-bond geometry (Å, °)

*D*—H⋯*A*	*D*—H	H⋯*A*	*D*⋯*A*	*D*—H⋯*A*
C16—H16*A*⋯O5^i^	0.93	2.57	3.389 (3)	147
C17—H17*A*⋯O2^ii^	0.93	2.70	3.614 (3)	170
C19—H19*A*⋯O1^iii^	0.93	2.59	3.428 (3)	150

Symmetry codes: (i) 
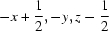
; (ii) 
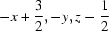
; (iii) 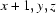.

**Fig. 1.**
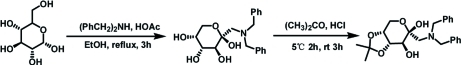
The synthesis path of title compound.

**Fig. 2.**
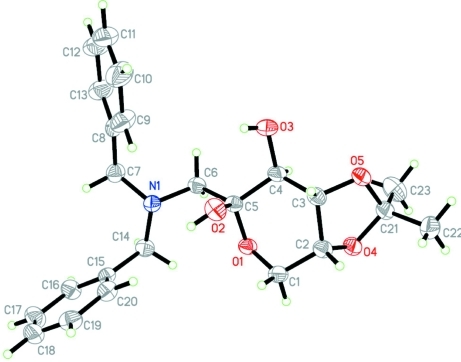
The molecular structure of the title compound with the atom numbering scheme. Displacement ellipsoids are drawn at 30% probability level. H atoms are presented as a small spheres of arbitrary radius.

**Fig. 3.**
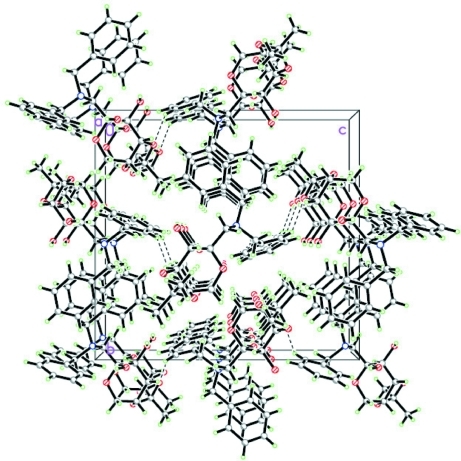
A view of the packing of title compound. Dashed lines indicate non–classical C—H···O hydrogen bonds.

**Fig. 4.**
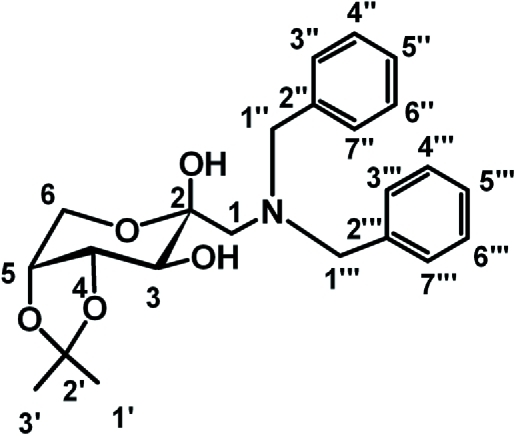
The structure of title compound, with atoms labeling corresponding to the characterization by NMR.

## supplementary crystallographic information

### Comment

Asymmetric epoxidation of olefins presents a powerful strategy for the synthesis of enriched epoxides. The title compound is a key intermediate for the preparation of an effective epoxidation catalyst which provides encouragingly high enantiomeric excess value for the epoxidation of *cis*–olefins and styrenes (Shu *et al.*, 2003; Tian *et al.*, 2000, 2002). Furthermore, it can be another starting material to synthesize the corresponding amino sugar derivatives.

The title compound is prepared *via* two steps including Amadori rearrangement and ketalization (Fig. 1). In the molecular structure of the title compound (Fig. 2), the fructopyranose ring adopts a chair conformation - torsion angles: C3–C2–C1–O1 = 37.9 (3)° and C4–C3–C2–C1 = -32.9 (3)°. The structure is stabilized by the non–classical intermolecular hydrogen bonds (Table 1, Fig. 3).

### Experimental

The synthesis of the title compound was shown in Fig. 1. The pure title compound was first obtained by chromatography. It (10 g) was recrystallized from a solution of ethyl ether (50 ml) cooling at 255 K to afford colourless crystals.

The molecule is characterized by NMR (Fig. 4). ^1^H NMR (400 MHz, CDCl_3_): δ 7.25–7.35 (10H, m, *Ar*–H), 4.15–4.18 (2H, m, H–5, H–6e), 3.99–4.07 (3H, m, H–1'', H–1''', H–4), 3.91 (1H, d, *J* = 13.2 Hz, H–6a), 3.48 (2H, d, *J* = 13.2 Hz, H–1'', H–1'''), 3.29 (1H, d, *J* = 7.2 Hz, H–3), 3.07 (1H, d, *J* = 13.2 Hz, H–1), 2.69 (1H, d, *J* = 13.2 Hz, H–1), 1.51 (3H, s, H–3'), 1.34 (3H, s, H–1').

^13^C NMR (101 MHz, CDCl_3_): δ 138.29 (C–2'', C–2'''), 129.31 (C–3'', C–7'', C–3''', C–7'''), 128.52 (C–4'', C–6'', C–4''', C–6'''), 127.46 (C–5'', C–5'''), 109.10 (C–2'), 96.21 (C–2), 77.79 (C–4), 73.68 (C–5), 72.17 (C–3), 59.18 (C–1'', C–1'''), 58.93 (C–6), 56.31 (C–1), 28.20 (C–1'), 26.28 (H–3').

HRMS(ES^+^): *m/z* [*M*+Cl]^-^ calcd. for C_23_H_29_NO_5_Cl: 434.1734; found: 434.1737.

### Refinement

All H atoms attached to C atoms were treated as riding, with C—H = 0.97Å for methylene group, C—H = 0.98Å for methyne group, C—H = 0.96Å for methyl group and C—H = 0.93Å for aryl, with *U*_iso_(H) = 1.5*U*_eq_(C) for the methyl groups and *U*_iso_(H) = 1.2*U*_eq_(C) for other. The hydroxyl H–atoms were refined as rigid, with O—H = 0.82Å and *U*_iso_(H) = 1.5*U*_eq_(O). An absolute structure could not be established reliably by anomalous scattering effects. The 1576 Friedel pairs were merged by "MERG 2" instruction of *SHELXL*.

### Crystal data

**Table:**

C_23_H_29_NO_5_	*D*_x_ = 1.232 Mg m^−^^3^
*M**_r_* = 399.47	Melting point: 363 K
Orthorhombic, *P*2_1_2_1_2_1_	Mo *K*α radiation, λ = 0.71073 Å
Hall symbol: P 2ac 2ab	Cell parameters from 2101 reflections
*a* = 8.328 (3) Å	θ = 2.5–22.9°
*b* = 15.635 (5) Å	µ = 0.09 mm^−^^1^
*c* = 16.547 (5) Å	*T* = 293 K
*V* = 2154.6 (12) Å^3^	Block, colourless
*Z* = 4	0.32 × 0.26 × 0.18 mm
*F*(000) = 856	

### Data collection

**Table:**

Bruker SMART APEX CCD diffractometer	3078 reflections with *I* > 2σ(*I*)
Radiation source: fine–focus sealed tube	*R*_int_ = 0.029
graphite	θ_max_ = 25.0°, θ_min_ = 2.5°
φ– and ω–scans	*h* = −9→9
8591 measured reflections	*k* = −12→18
3746 independent reflections	*l* = −19→19

### Refinement

**Table:**

Refinement on *F*^2^	Secondary atom site location: difference Fourier map
Least-squares matrix: full	Hydrogen site location: inferred from neighbouring sites
*R*[*F*^2^ > 2σ(*F*^2^)] = 0.037	H-atom parameters constrained
*wR*(*F*^2^) = 0.099	*w* = 1/[σ^2^(*F*_o_^2^) + (0.0586*P*)^2^] where *P* = (*F*_o_^2^ + 2*F*_c_^2^)/3
*S* = 0.99	(Δ/σ)_max_ < 0.001
3746 reflections	Δρ_max_ = 0.16 e Å^−^^3^
264 parameters	Δρ_min_ = −0.14 e Å^−^^3^
12 restraints	Extinction correction: *SHELXL*, Fc^*^=kFc[1+0.001xFc^2^λ^3^/sin(2θ)]^-1/4^
Primary atom site location: structure-invariant direct methods	Extinction coefficient: 0.0109 (15)

### Special details

**Table:**

Geometry. All s.u.'s (except the s.u. in the dihedral angle between two l.s. planes) are estimated using the full covariance matrix. The cell s.u.'s are taken into account individually in the estimation of s.u.'s in distances, angles and torsion angles; correlations between s.u.'s in cell parameters are only used when they are defined by crystal symmetry. An approximate (isotropic) treatment of cell s.u.'s is used for estimating s.u.'s involving l.s. planes.
Refinement. Refinement of *F*^2^ against ALL reflections. The weighted *R*–factor *wR* and goodness of fit *S* are based on *F*^2^, conventional *R*–factors *R* are based on *F*, with *F* set to zero for negative *F*^2^. The threshold expression of *F*^2^ > σ(*F*^2^) is used only for calculating *R*–factors(gt) *etc*. and is not relevant to the choice of reflections for refinement. *R*–factors based on *F*^2^ are statistically about twice as large as those based on *F*, and *R*–factors based on ALL data will be even larger.

### Fractional atomic coordinates and isotropic or equivalent isotropic displacement parameters (Å^2^)

**Table:**

	*x*	*y*	*z*	*U*_iso_*/*U*_eq_	
N1	0.45371 (19)	−0.03531 (9)	−0.03276 (9)	0.0445 (4)	
O1	0.26735 (16)	0.12669 (8)	0.01447 (7)	0.0462 (3)	
O2	0.44619 (18)	0.05470 (9)	0.09987 (8)	0.0570 (4)	
H2A	0.5176	0.0562	0.0658	0.086*	
O3	0.1840 (3)	−0.02563 (10)	0.17613 (11)	0.0803 (5)	
H3	0.2784	−0.0405	0.1776	0.120*	
O4	0.03036 (19)	0.23207 (9)	0.11486 (7)	0.0552 (4)	
O5	0.02593 (19)	0.14018 (9)	0.22165 (8)	0.0597 (4)	
C1	0.2928 (3)	0.20454 (12)	0.05737 (11)	0.0531 (5)	
H1A	0.2674	0.2522	0.0221	0.064*	
H1B	0.4054	0.2088	0.0718	0.064*	
C2	0.1933 (3)	0.21137 (12)	0.13278 (11)	0.0496 (5)	
H2B	0.2389	0.2553	0.1682	0.059*	
C3	0.1735 (3)	0.12850 (13)	0.17977 (11)	0.0488 (5)	
H3B	0.2610	0.1232	0.2190	0.059*	
C4	0.1688 (3)	0.04895 (12)	0.12720 (11)	0.0494 (5)	
H4A	0.0637	0.0469	0.1007	0.059*	
C5	0.2971 (2)	0.05151 (12)	0.06144 (10)	0.0430 (4)	
C6	0.2938 (2)	−0.02364 (12)	0.00288 (11)	0.0468 (5)	
H6A	0.2621	−0.0752	0.0313	0.056*	
H6B	0.2159	−0.0129	−0.0395	0.056*	
C7	0.4851 (3)	−0.12466 (12)	−0.05526 (12)	0.0552 (5)	
H7A	0.5860	−0.1272	−0.0844	0.066*	
H7B	0.4012	−0.1435	−0.0918	0.066*	
C8	0.4931 (3)	−0.18588 (12)	0.01473 (13)	0.0530 (5)	
C9	0.5592 (4)	−0.16474 (16)	0.08772 (15)	0.0766 (7)	
H9A	0.5991	−0.1098	0.0956	0.092*	
C10	0.5678 (4)	−0.2241 (2)	0.15046 (17)	0.0973 (10)	
H10A	0.6114	−0.2084	0.2000	0.117*	
C11	0.5121 (4)	−0.30535 (19)	0.1390 (2)	0.0940 (10)	
H11A	0.5185	−0.3452	0.1806	0.113*	
C12	0.4479 (4)	−0.32744 (19)	0.0676 (3)	0.0989 (10)	
H12A	0.4106	−0.3829	0.0598	0.119*	
C13	0.4368 (3)	−0.26813 (14)	0.00508 (19)	0.0782 (7)	
H13A	0.3909	−0.2842	−0.0438	0.094*	
C14	0.4789 (2)	0.02062 (13)	−0.10350 (11)	0.0487 (5)	
H14A	0.4304	0.0759	−0.0929	0.058*	
H14B	0.4248	−0.0041	−0.1499	0.058*	
C15	0.6535 (2)	0.03325 (11)	−0.12364 (10)	0.0422 (4)	
C16	0.7148 (3)	0.01268 (14)	−0.19830 (12)	0.0573 (5)	
H16A	0.6475	−0.0112	−0.2371	0.069*	
C17	0.8741 (3)	0.02674 (16)	−0.21685 (14)	0.0697 (7)	
H17A	0.9137	0.0117	−0.2674	0.084*	
C18	0.9742 (3)	0.06300 (15)	−0.16054 (15)	0.0674 (6)	
H18A	1.0814	0.0729	−0.1731	0.081*	
C19	0.9159 (3)	0.08463 (14)	−0.08581 (15)	0.0604 (6)	
H19A	0.9832	0.1094	−0.0476	0.072*	
C20	0.7577 (3)	0.06950 (12)	−0.06777 (12)	0.0525 (5)	
H20A	0.7192	0.0839	−0.0168	0.063*	
C21	−0.0606 (3)	0.21023 (14)	0.18498 (12)	0.0547 (5)	
C22	−0.0670 (4)	0.28284 (16)	0.24500 (15)	0.0808 (8)	
H22A	0.0402	0.2995	0.2592	0.121*	
H22B	−0.1232	0.2646	0.2926	0.121*	
H22C	−0.1221	0.3306	0.2213	0.121*	
C23	−0.2231 (3)	0.1807 (2)	0.15849 (16)	0.0861 (8)	
H23A	−0.2117	0.1346	0.1207	0.129*	
H23B	−0.2791	0.2273	0.1332	0.129*	
H23C	−0.2829	0.1614	0.2046	0.129*	

### Atomic displacement parameters (Å^2^)

**Table:**

	*U*^11^	*U*^22^	*U*^33^	*U*^12^	*U*^13^	*U*^23^
N1	0.0465 (10)	0.0434 (8)	0.0437 (8)	0.0012 (7)	0.0014 (7)	0.0021 (7)
O1	0.0531 (8)	0.0446 (7)	0.0408 (6)	−0.0049 (6)	0.0027 (6)	0.0049 (6)
O2	0.0469 (8)	0.0727 (9)	0.0514 (7)	−0.0011 (7)	−0.0076 (6)	0.0009 (7)
O3	0.1109 (15)	0.0558 (9)	0.0741 (10)	0.0149 (9)	0.0334 (11)	0.0294 (8)
O4	0.0653 (10)	0.0561 (8)	0.0442 (7)	0.0055 (7)	0.0080 (7)	0.0095 (6)
O5	0.0731 (10)	0.0584 (9)	0.0475 (7)	0.0065 (8)	0.0185 (7)	0.0122 (7)
C1	0.0606 (14)	0.0458 (10)	0.0528 (11)	−0.0090 (9)	0.0062 (10)	0.0019 (9)
C2	0.0558 (14)	0.0499 (11)	0.0430 (10)	−0.0093 (9)	0.0012 (10)	−0.0005 (9)
C3	0.0522 (12)	0.0571 (11)	0.0370 (9)	−0.0009 (10)	0.0017 (9)	0.0052 (9)
C4	0.0547 (13)	0.0472 (10)	0.0463 (10)	0.0015 (9)	0.0048 (10)	0.0144 (9)
C5	0.0411 (11)	0.0450 (9)	0.0431 (9)	−0.0026 (8)	−0.0022 (8)	0.0054 (8)
C6	0.0438 (12)	0.0464 (10)	0.0501 (10)	−0.0039 (8)	−0.0001 (9)	0.0051 (9)
C7	0.0651 (14)	0.0494 (11)	0.0511 (10)	0.0006 (10)	0.0012 (10)	−0.0058 (9)
C8	0.0459 (12)	0.0477 (11)	0.0655 (13)	0.0076 (9)	0.0075 (10)	0.0001 (10)
C9	0.105 (2)	0.0565 (13)	0.0679 (14)	0.0125 (14)	−0.0144 (15)	0.0049 (12)
C10	0.129 (3)	0.090 (2)	0.0731 (16)	0.0303 (19)	−0.0086 (17)	0.0177 (16)
C11	0.093 (2)	0.0794 (19)	0.110 (2)	0.0139 (16)	0.014 (2)	0.0413 (18)
C12	0.080 (2)	0.0638 (16)	0.153 (3)	−0.0102 (15)	0.000 (2)	0.0341 (19)
C13	0.0732 (17)	0.0566 (14)	0.1049 (19)	−0.0096 (12)	−0.0098 (15)	0.0108 (14)
C14	0.0523 (13)	0.0525 (10)	0.0413 (9)	−0.0004 (10)	−0.0045 (9)	0.0049 (9)
C15	0.0498 (12)	0.0368 (9)	0.0400 (9)	0.0008 (8)	−0.0027 (9)	0.0020 (8)
C16	0.0625 (15)	0.0628 (12)	0.0466 (11)	−0.0070 (11)	0.0016 (10)	−0.0054 (10)
C17	0.0740 (17)	0.0777 (16)	0.0575 (12)	−0.0052 (13)	0.0190 (12)	−0.0024 (13)
C18	0.0500 (14)	0.0664 (14)	0.0857 (16)	0.0005 (11)	0.0078 (13)	0.0138 (13)
C19	0.0523 (14)	0.0537 (12)	0.0752 (15)	−0.0066 (10)	−0.0172 (12)	0.0066 (11)
C20	0.0599 (14)	0.0515 (11)	0.0461 (10)	0.0015 (10)	−0.0060 (10)	−0.0040 (9)
C21	0.0632 (14)	0.0564 (11)	0.0444 (10)	0.0050 (10)	0.0082 (10)	0.0064 (9)
C22	0.112 (2)	0.0641 (14)	0.0663 (14)	0.0090 (15)	0.0213 (16)	−0.0049 (13)
C23	0.0639 (18)	0.123 (2)	0.0719 (15)	−0.0073 (16)	0.0027 (14)	0.0065 (16)

### Geometric parameters (Å, °)

**Table:**

N1—C6	1.468 (2)	C9—H9A	0.9300
N1—C7	1.469 (2)	C10—C11	1.366 (5)
N1—C14	1.476 (2)	C10—H10A	0.9300
O1—C1	1.425 (2)	C11—C12	1.343 (5)
O1—C5	1.431 (2)	C11—H11A	0.9300
O2—C5	1.396 (2)	C12—C13	1.392 (4)
O2—H2A	0.8200	C12—H12A	0.9300
O3—C4	1.425 (2)	C13—H13A	0.9300
O3—H3	0.8200	C14—C15	1.505 (3)
O4—C2	1.426 (3)	C14—H14A	0.9700
O4—C21	1.427 (2)	C14—H14B	0.9700
O5—C3	1.423 (3)	C15—C16	1.375 (3)
O5—C21	1.445 (2)	C15—C20	1.389 (3)
C1—C2	1.502 (3)	C16—C17	1.380 (3)
C1—H1A	0.9700	C16—H16A	0.9300
C1—H1B	0.9700	C17—C18	1.373 (3)
C2—C3	1.520 (3)	C17—H17A	0.9300
C2—H2B	0.9800	C18—C19	1.371 (3)
C3—C4	1.518 (3)	C18—H18A	0.9300
C3—H3B	0.9800	C19—C20	1.372 (3)
C4—C5	1.526 (3)	C19—H19A	0.9300
C4—H4A	0.9800	C20—H20A	0.9300
C5—C6	1.523 (3)	C21—C23	1.495 (4)
C6—H6A	0.9700	C21—C22	1.509 (3)
C6—H6B	0.9700	C22—H22A	0.9600
C7—C8	1.504 (3)	C22—H22B	0.9600
C7—H7A	0.9700	C22—H22C	0.9600
C7—H7B	0.9700	C23—H23A	0.9600
C8—C9	1.368 (3)	C23—H23B	0.9600
C8—C13	1.378 (3)	C23—H23C	0.9600
C9—C10	1.394 (4)		
			
C6—N1—C7	112.42 (15)	C10—C9—H9A	119.4
C6—N1—C14	111.94 (15)	C11—C10—C9	119.9 (3)
C7—N1—C14	109.70 (15)	C11—C10—H10A	120.0
C1—O1—C5	113.91 (13)	C9—C10—H10A	120.0
C5—O2—H2A	109.5	C12—C11—C10	119.8 (3)
C4—O3—H3	109.5	C12—C11—H11A	120.1
C2—O4—C21	106.38 (15)	C10—C11—H11A	120.1
C3—O5—C21	108.89 (14)	C11—C12—C13	120.6 (3)
O1—C1—C2	113.12 (16)	C11—C12—H12A	119.7
O1—C1—H1A	109.0	C13—C12—H12A	119.7
C2—C1—H1A	109.0	C8—C13—C12	120.9 (3)
O1—C1—H1B	109.0	C8—C13—H13A	119.6
C2—C1—H1B	109.0	C12—C13—H13A	119.6
H1A—C1—H1B	107.8	N1—C14—C15	112.98 (15)
O4—C2—C1	111.62 (16)	N1—C14—H14A	109.0
O4—C2—C3	101.33 (16)	C15—C14—H14A	109.0
C1—C2—C3	115.10 (17)	N1—C14—H14B	109.0
O4—C2—H2B	109.5	C15—C14—H14B	109.0
C1—C2—H2B	109.5	H14A—C14—H14B	107.8
C3—C2—H2B	109.5	C16—C15—C20	117.5 (2)
O5—C3—C4	111.21 (17)	C16—C15—C14	121.80 (18)
O5—C3—C2	103.50 (16)	C20—C15—C14	120.65 (17)
C4—C3—C2	114.08 (14)	C15—C16—C17	121.3 (2)
O5—C3—H3B	109.3	C15—C16—H16A	119.3
C4—C3—H3B	109.3	C17—C16—H16A	119.3
C2—C3—H3B	109.3	C18—C17—C16	119.9 (2)
O3—C4—C3	110.03 (15)	C18—C17—H17A	120.0
O3—C4—C5	111.37 (16)	C16—C17—H17A	120.0
C3—C4—C5	111.65 (16)	C19—C18—C17	120.0 (2)
O3—C4—H4A	107.9	C19—C18—H18A	120.0
C3—C4—H4A	107.9	C17—C18—H18A	120.0
C5—C4—H4A	107.9	C18—C19—C20	119.6 (2)
O2—C5—O1	111.86 (14)	C18—C19—H19A	120.2
O2—C5—C6	109.49 (15)	C20—C19—H19A	120.2
O1—C5—C6	106.55 (13)	C19—C20—C15	121.7 (2)
O2—C5—C4	107.41 (15)	C19—C20—H20A	119.1
O1—C5—C4	106.71 (15)	C15—C20—H20A	119.1
C6—C5—C4	114.87 (16)	O4—C21—O5	104.94 (17)
N1—C6—C5	109.56 (15)	O4—C21—C23	108.43 (18)
N1—C6—H6A	109.8	O5—C21—C23	109.9 (2)
C5—C6—H6A	109.8	O4—C21—C22	111.94 (18)
N1—C6—H6B	109.8	O5—C21—C22	108.14 (18)
C5—C6—H6B	109.8	C23—C21—C22	113.1 (2)
H6A—C6—H6B	108.2	C21—C22—H22A	109.5
N1—C7—C8	114.70 (15)	C21—C22—H22B	109.5
N1—C7—H7A	108.6	H22A—C22—H22B	109.5
C8—C7—H7A	108.6	C21—C22—H22C	109.5
N1—C7—H7B	108.6	H22A—C22—H22C	109.5
C8—C7—H7B	108.6	H22B—C22—H22C	109.5
H7A—C7—H7B	107.6	C21—C23—H23A	109.5
C9—C8—C13	117.7 (2)	C21—C23—H23B	109.5
C9—C8—C7	122.96 (19)	H23A—C23—H23B	109.5
C13—C8—C7	119.3 (2)	C21—C23—H23C	109.5
C8—C9—C10	121.2 (3)	H23A—C23—H23C	109.5
C8—C9—H9A	119.4	H23B—C23—H23C	109.5
			
C3—C2—C1—O1	37.9 (3)	C4—C3—C2—C1	−32.9 (3)

### Hydrogen-bond geometry (Å, °)

**Table:**

*D*—H···*A*	*D*—H	H···*A*	*D*···*A*	*D*—H···*A*
C16—H16A···O5^i^	0.93	2.57	3.389 (3)	147
C17—H17A···O2^ii^	0.93	2.70	3.614 (3)	170
C19—H19A···O1^iii^	0.93	2.59	3.428 (3)	150

Symmetry codes: (i) −*x*+1/2, −*y*, *z*−1/2; (ii) −*x*+3/2, −*y*, *z*−1/2; (iii) *x*+1, *y*, *z*.
